# Proteomic characterization of *Toxoplasma gondii* ME49 derived strains resistant to the artemisinin derivatives artemiside and artemisone implies potential mode of action independent of ROS formation

**DOI:** 10.1016/j.ijpddr.2022.11.005

**Published:** 2022-12-08

**Authors:** Joachim Müller, Carling Schlange, Manfred Heller, Anne-Christine Uldry, Sophie Braga-Lagache, Richard K. Haynes, Andrew Hemphill

**Affiliations:** a Institute of Parasitology, University of Bern, Department of Infectious Diseases and Pathobiology, Vetsuisse Faculty, Länggass-Strasse 122, CH-3012, Bern, Switzerland; b Proteomics & Mass Spectrometry Core Facility, Department for BioMedical Research (DBMR), University of Bern, Freiburgstrasse 15, CH-3010, Bern, Switzerland; c Centre of Excellence for Pharmaceutical Sciences, Faculty of Health Sciences, North‐West University, Potchefstroom, 2520, South Africa

**Keywords:** Apicomplexan parasites, Drug resistance, Mass spectrometry, Model organism, Reactive oxygen species, Untargeted proteomics

## Abstract

The sesquiterpene lactone artemisinin and its amino-artemisinin derivatives artemiside (GC008) and artemisone (GC003) are potent antimalarials. The mode of action of artemisinins against *Plasmodium* sp is popularly ascribed to 'activation' of the peroxide group by heme-Fe(II) or labile Fe(II) to generate C-radicals that alkylate parasite proteins. An alternative postulate is that artemisinins elicit formation of reactive oxygen species by interfering with flavin disulfide reductases resposible for maintaining intraparasitic redox homeostasis. However, in contradistinction to the heme-activation mechanism, the amino-artemisinins are effective *in vitro* against non-heme-degrading apicomplexan parasites including *T. gondii*, with IC _50_ values of 50–70 nM, and induce distinct ultrastructural alterations. However, *T. gondii* strains readily adapted to increased concentrations (2.5 μM) of these two compounds within few days. Thus, *T. gondii* strains that were resistant against artemisone and artemiside were generated by treating the *T. gondii* reference strain ME49 with stepwise increasing amounts of these compounds, yielding the artemisone resistant strain GC003^R^ and the artemiside resistant strain GC008^R^. Differential analyses of the proteomes of these resistant strains compared to the wildtype ME49 revealed that 215 proteins were significantly downregulated in artemisone resistant tachyzoites and only 8 proteins in artemiside resistant tachyzoites as compared to their wildtype. Two proteins, namely a hypothetical protein encoded by ORF TGME49_236950, and the rhoptry neck protein RON2 encoded by ORF TGME49_300100 were downregulated in both resistant strains. Interestingly, eight proteins involved in ROS scavenging including catalase and superoxide dismutase were amongst the differentially downregulated proteins in the artemisone-resistant strain. In parallel, ROS formation was significantly enhanced in isolated tachyzoites from the artemisone resistant strain and – to a lesser extent – in tachyzoites from the artemiside resistant strain as compared to wildtype tachyzoites. These findings suggest that amino-artemisinin derivatives display a mechanism of action in *T. gondii* distinct from *Plasmodium*.

## Graphical abstract

**Figure:**
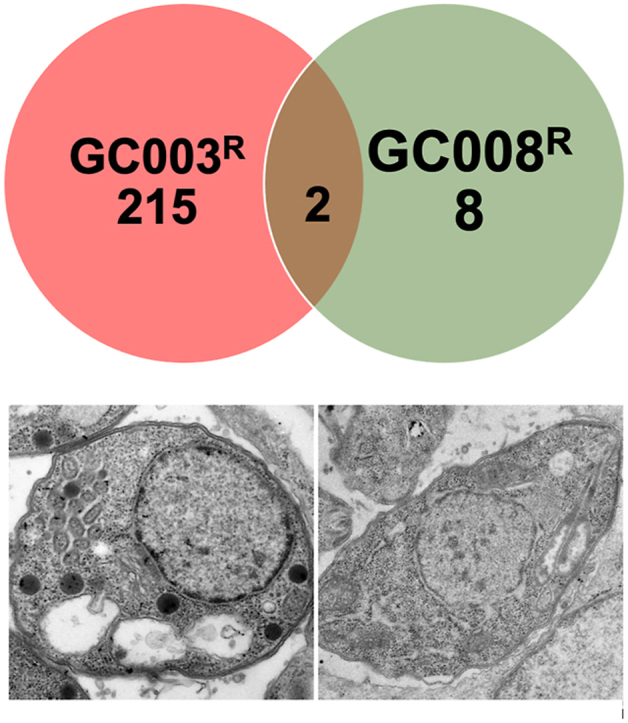

## 1 Introduction

*T. gondii* is one of the most widespread parasites worldwide, in most cases causing asymptomatic infections (Dubey, 2010). In some cases, e.g. in immunocompromised patients and in fetuses, symptomatic infections, i.e. toxoplasmosis, characterized by proliferating tachyzoites, may occur (Dunay et al., 2018). *T. gondii* belongs to the phylum Apicomplexa, which is associated with the kingdom Alveolata of the superkingdom SAR (Adl et al., 2012). The life cycle comprises three stages, namely tachyzoites, bradyzoites and sporozoites within oocysts (Attias et al., 2020). Tachyzoites proliferate in various cell lines and are accessible to functional genetic manipulations (Limenitakis and Soldati-Favre, 2011). Moreover, the genome of various strains has been sequenced (see www.toxodb.org). *T. gondii* can thus be considered as a well-established model system to study various aspects of the biology of intracellular protozoan parasites.

**Fig. 1:**
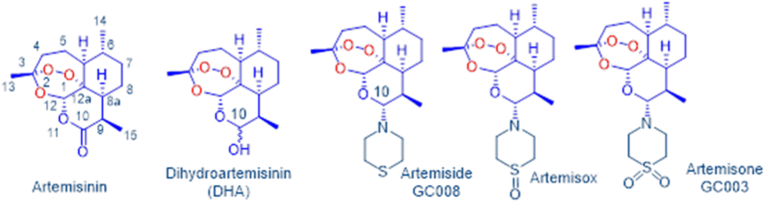
The sesquiterpene lactone artemisinin originally isolated from the Traditional Chinese Medicinal herb *Artemisia annua* and its derivatives such as DHA contain an active peroxide pharmacophore and are used for treatment of malaria. The amino-artemisinins artemiside (GC008), artemisox and artemisone **(**GC003) in contradistinction to current derivatives possess a nitrogen atom attached to C-10 that enhances biological activities. The sulfide artemiside is rapidly metabolized via the sulfoxide artemisox into the sulfone artemisone.

One of these aspects is the mode of action of antiparasitic drugs. The availability of a plethora of effective compounds against the protozoan parasite with the highest impact on health, *Plasmodium* sp., has paved the way to numerous drug repurposing studies investigating the effects of antimalarials against *T. gondii* and other parasites (Van Voorhis et al., 2016). Initial screening of these compounds is facilitated by the fact that strains expressing easy-to-use markers such as *Escherichia. coli* beta-galactosidase exist for *T. gondii* (Seeber and Boothroyd, 1996) as well as for the closely related *Nesopora caninum* (Müller et al., 2017). One such antimalarial class of drug eminently suitable for repurposing are the artemisinins (van Agtmael et al., 1999; Cui and Su, 2009). Identified in the early 1970s as the active principle of the Chinese medicinal herb *Artemisia annua* traditionally used as an anti-pyretic, the parent drug artemisinin is a sesquiterpene lactone that uniquely contains the peroxide group as the active pharmacophore (Chaturvedi et al., 2010) (Fig. 1). Several derivatives derived from the reduction product of artemisinin, dihydroartemisinin (DHA; Fig. 1), are widely used as antimalarial drugs (Tilley et al., 2016). Activities of these artemisinins against *T. gondii* (Berens et al., 1998; Dunay et al., 2009) and *N. caninum* (Kim et al., 2002; Mazuz et al., 2012; Müller et al., 2015b) have ben documented. In our own work on new artemisinin derivatives for malaria and other indications, we have prepared structurally distinct derivatives from DHA bearing an amino group at C-10 that we refer to as amino-artemisinins (Fig. 1). One such derivative is artemiside (GC008), which is rapidly metabolized (Haynes et al., 2006) into the sulfoxide artemisox and thence into the sulfone artemisone (GC003). All three drugs in their own right are potently active against malaria *in vitro* both against asexual (IC_50_ = 1.1–1.95 nM) and sexually differentiated *P. falciparum* blood stage parasites (IC_50_ 1.5–42.4 nM) (Gibhard et al., 2021).

The antimalarial mechanism of action (MoA) of artemisinins is open to debate. The thesis involving 'activation' by ferrous heme [heme-Fe(II)] appears to be the most strongly supported for *Plasmodium* blood stream forms (Giannangelo et al., 2019; Quadros et al., 2022). ‘Activation' is presumed to be followed by generation of C-centred radicals that alkylate parasite proteins. The difficulties in this thesis have been thoroughly discussed elsewhere (Haynes et al., 2013). It has also been shown that artemisinins and synthetic analogues rapidly oxidize reduced flavin cofactors of the disulfide reductases glutathione reductase, thioredoxin reductase and others important for maintaining redox homeostasis in the malaria parasite (Haynes et al., 2010, 2012). The resulting oxidative stress may generate reactive oxygen species (ROS), shown to accompany treatment with artemisinins, in particular in rat hepatocytes (Leskovac and Peggins, 1992) and yeast (Wang et al., 2010). In a more recent study using genome-wide CRISPR screens, DHA susceptibility in *T. gondii* has again been linked to heme metabolism, without, however involving alterations in ROS formation (Harding et al., 2020). Another study based on the analysis of CRISPR induced point-mutations in *T. gondii* reveales other potential mechanism of resistance to artemisinin, which are not linked to heme or ROS formation (Rosenberg et al., 2019).

Be this as it may, a suitable manner to elucidate the mechanism of action is to conduct analyses of parasite strains that are resistant to amino-artemisinin derivatives.

Here, we present results obtained from proteomic and phenotypic studies comparing *T. gondii* strains that have adapted to artemisone or artemiside to their wildtype strain ME49 suggesting indeed that mechanisms other than stimulation of ROS formation may be responsible for the mode of action of these amino-artemisinin derivatives.

## 2 Materials and methods

### 2.1 Chemicals

If not otherwise stated, all biochemical reagents were from Sigma (St Louis, MO, USA). Culture media were purchased from Thermo Fisher Scientific (Waltham, MA, USA). Reference compounds, artemiside (GC008) and artemisone (GC003) used for screening were ≥98% pure as established previously (Coertzen et al., 2018; Watson et al., 2021). 10 mM stock solutions were prepared in dimethyl sulfoxide (DMSO) and stored at −20 °C. The structures are depicted in Fig. 1.

### 2.2 Culture of host cells and parasites

Human foreskin fibroblasts (HFF, commercially available from ATCC, PCS-201-010) were maintained in Dulbecco's Minimal Essential Medium (DMEM) containing 10% fetal calf serum at 37 °C/5% CO_2_. The use of HFF from this source did not require the study to be reviewed or approved by an ethics committee, as the sources were non-identifieable and cultures were commercially available. Tachyzoites of *T. gondii* ME49 and *T. gondii* RH strain expressing beta-galacosidase (Seeber and Boothroyd, 1996) were maintained on HFF and sub-cultivated by serial passages. Tachyzoites were harvested by removal of the cell layer with a cell scraper, isolated and purified by pressing the cell suspension through a polycarbonate membrane (3 μm pore size) (Ramseier et al., 2021).

### 2.3 *In vitro* assessments of drug susceptibility in parasites and host cells

Drug efficacy tests using a *T. gondii* RH strain expressing beta-galactosidase were performed in 96-well-plates with confluent HFFs as host cells as described earlier (McFadden et al., 1997; Müller et al., 2015a; Basto et al., 2019). Serial dilutions of artemisone and artemiside (0–1 μM) were prepared in culture medium and added either 5 min before infecting the host cells or 3 h after infection. Host cell toxicity against uninfected HFFs was determined as described (Müller et al., 2020).

To determine susceptibilities of strain ME49 and resistant strains derived from this wildtype, HFF were grown in 6-well plates until a confluent monolayer was formed. Just prior to infection, dilutions of artemisone and artemiside (0–5 μM) were added. Controls received the corresponding amounts of DMSO. HFF were then infected with 5 × 10^4^ freshly purified tachyzoites, in a total volume of 5 ml. After four days, cells were collected with a cell scraper, centrifuged, washed once in PBS, and the pellet was stored at −20 °C prior to quantification of parasite proliferation. After DNA extraction using the Nucleospin Rapid lyse kit (Macherey-Nagel, Düren, Germany) according to the manufacturer's instructions, tachyzoites were quantified by *Toxoplasma gondii*-specific quantitative PCR as described (Costa et al., 2000). Concentrations corresponding to 50% inhibition as compared to the controls were calculated using the logit-log algorithm as described (Müller and Hemphill, 2013). assays were performed in triplicates.

Minimal inhibitory concentrations (MICs) were determined in 96-well-plates using serial dilutions by a factor 2 of the compounds starting at 25 μM using an inoculum of 10^3^ freshly purified tachyzoites. After five days, the MICs were determined by observing the wells under the microscope starting from higher to lower concentrations. The first concentration at which rosettes were visible is given as the MIC.

### 2.4 *In vitro* assessments of reactive oxygen species formation

The formation of reactive oxygen species (ROS) was assayed using dichlorofluorescein-diacetate (DCF-DA) as described previously (Yazdani, 2015). Briefly, isolated tachyzoites separated from host cell debris, or HFF infected with tachyzoites as described above, were suspended in phosphate-buffered saline (PBS) containing 10 mM 2-[4-(2-hydroxyethyl)piperazin-1-yl]ethanesulfonic acid (HEPES) and distributed into 96-well-plates in the presence of DMSO or compounds to be tested. FeCl_2_ (5 μM) yielding Fe(II) ions in solution was included as a positive control. The reaction was started by adding DCF-DA (10 μM final concentration). At various time points, fluorescence was measured (excitation at 485 nm, emission at 535 nm) using a Hidex Sense microplate reader (Hidex, Turku, Finland).

### 2.5 Transmission electron microscopy (TEM)

For TEM studies, T25 flasks containing HFF monolayers infected with *T. gondii* tachyzoites were exposed to 1 μM artemiside (GC008), artemisone (GC003) or the corresponding amounts of DMSO, and were cultured at 37 °C/5% CO2. Fixation of samples was carried out after 6, 24, and 48 h after initiation of drug treatments. For this, monolayers were washed in 0.1 M Na-cacodylate pH 7.3 and subsequently immersed in cacodylate buffer containing 2% glutaraldehyde, scraped from the culture flask, and they were further fixed for 1–2 h at room temperature or overnight at 4 °C. After 3 washes in cacodylate buffer, post-fixation was carried out in cacodylate buffer containing 2% osmium tetroxide. After three washes in H_2_O, specimens were pre-stained in UranyLess (Electron Microscopy Science, Hatfield, PA, USA), and dehydrated stepwise in a graded series of ethanol (30-50-70-90 and 3 × 100%). Following dehydration, samples were embedded in Epon812 epoxy resin as described (Anghel et al., 2021b). Ultrathin sections were cut using an ultramicrotome (Reichert & Jung, Vienna, Austria), sections were loaded onto 300 mesh formvar/carbon coated nickel grids (Plano GmbH, Wetzlar, Germany), and specimens were viewed on a CM12 TEM operating at 80 kV.

### 2.6 Long term treatments and generation of resistant strains *T. gondii_*GC003^R^ and *T. gondii*_GC008^R^

T25 culture flasks were seeded with 5 × 10^5^ HFF and cultures were maintained for 4 days at 37 °C/5% CO_2_. On day 0, the monolayers were infected with 5 × 10^5^ *T. gondii* ME49 tachyzoites. At 4.5 h post infection (p.i.) the medium was removed and the flasks were washed twice with Hank's Balanced Salt Solution (HBSS) and fresh medium containing either artemiside (GC008) or artemisone (GC003), both at 1 μM, or DMSO (0.25%) as a negative control, were added. Cultures were monitored microscopically on a daily basis, and once parasite proliferation was noted, the drug concentration was increased to 2.5 μM (for artemisone) and up to 5 μM (for artemiside). Overall treatments lasted 29 days, with medium changes and addition of fresh compounds approximatively every 3–4 days. Wildtype parasites were maintained in parallel in the presence of 0.25% DMSO. Whenever parasites started to lyse the HFF monolayer, they were passaged onto a new T25 flask containing a confluent layer of host cells. Cultures were continuously observed by light microscopy. On day 29, continued proliferation of tachyzoites was observed in the presence of both drugs, suggestive for resistance formation. The drug in the flasks was exchanged with DMEM without compound, and after 24 h, the two resistant strains *T. gondii*_GC003^R^ and *T. gondii*_GC008^R^, as well as the wildtype strain *T. gondii* ME49 were either frozen down as stabilates, or were transferred into Vero cell culture for subsequent proteomic analysis.

### 2.7 Proteomic analysis of resistant strains

Prior to proteomic analysis of *T. gondii* GC003^R^, *T. gondii* GC008^R^ and *T. gondii* WT tachyzoites, parasites were cultured in Vero cells in T175 culture flasks in the absence of drugs and subcultured twice. To liberate tachyzoites from host cells, infected cells were repeatedly passed through a syringe, and tachyzoites were subsequently passed through a polycarbonate membrane (3 μm pore size) and stored on ice. Parasites were counted and were centrifuged at 800×*g* for 10 min at 4 °C. The supernatant was removed and each of the 3 pellets was resuspended in 1.5 ml DMEM, and divided into three aliquotes of 500 μL each. Following another centrifugation step, the pellets were stored at −80 °C for subsequent whole-cell shotgun mass spectrometry. For each strain, two technical and three biological replicates were analyzed as follows. Cell pellets were lysed in 100 μL 8M urea/100 mM Tris/HCl pH 8, reduced with 10 mM DTT for 30 min at 37 °C, alkylated with 50 mM iodoacetamide for 30 min in the dark, and proteins precipitated with acetone at −20 °C overnight. The pellet was re-suspended in 50 μL 8M urea in 50 mM Tris/HCl pH8 and protein concentration was determinate with Qubit Protein Assay (Invitrogen). The urea concentration was reduced to 4 mM by dilution with 20 mM Tris/HCl, 2 mM CaCl_2_ and an aliquot corresponding to 10 μg protein was digested with sequencing grade LysC for 2 h at 37 °C followed by urea dilution to 1.6 M and sequencing grade trypsin (Promega) at room temperature overnight. Protease to protein ratio (w/w) was 1:50 and digestions were stopped with TFA at a final concentration of 1% (v/v). The digests were analyzed by liquid chromatography on an Ultimate 3000 coupled to a orbitrap LUMOS mass spectrometer (Thermo Fischer, Bremen; Germany) with two injections of 500 ng peptides. Samples were loaded in random order onto a pre-column (C18 PepMap 100, 5 μm, 100A, 300 μm i.d. x 5 mm length) at a flow rate of 10 μL/min with solvent C (0.05% TFA in water/acetonitrile 98:2). After loading, peptides were eluted in back flush mode onto a C18 column (3 μm, 100 Å, 75 μm × 15 cm, Nikkyo Technos, Tokyo, Japan) by applying a 90-min gradient of 5% acetonitrile to 40% in water, 0.1% formic acid, at a flow rate of 400 nl/min. Data acquisition was made in data dependent mode with precursor ion scans recorded in the orbitrap with resolution of 120′000 (at m/z = 250) parallel to top speed fragment spectra of the most intense precursor ions in the Linear trap for a cycle time of 3 s maximum.

Mass spectrometry data was processed by MaxQuant software, version 1.6.14.0, against the ToxoDB-47_TgondiiME49_AnnotatedProteins fasta protein sequence database without matching between runs option. Strict trypsin cleavage rule was applied, allowing for up to three missed cleavages, variable modifications of protein N-terminal acetylation and oxidation of methionine, static modification of cysteine with carbamidomethylation. Precursor and fragment mass tolerances were set to 10 ppm and 0.4 Da, respectively. Peptide spectrum matches, peptide and protein group identifications were filtered to a 1% false discovery rate (FDR) based on reversed database sequence matches, and a minimum of two razor or unique peptides were required to accept a protein group identification. Protein identifications considered as contaminations (e.g. trypsin or BSA) as well as proteins identified only by site were removed for statistical validation. Differential protein abundance tests between the different sample groups were done with LFQ and Top3 protein intensities as described elsewhere (Uldry et al., 2022).

### 2.8 Structural model and protein interaction networks

Structural modeling of ORF TgME49_236950 was performed using the Swiss Model homology modelling tools (Waterhouse et al., 2018) of the Swiss Model repository (Bienert et al., 2017). Protein-protein interaction networks were generated using the STRING knowledge base and software tool (https://string-db.org/).

## 3 Results

### 3.1 Artemisone (GC003) and artemiside (GC008) inhibit *T. gondii* proliferation *in vitro*

**Fig. 2:**
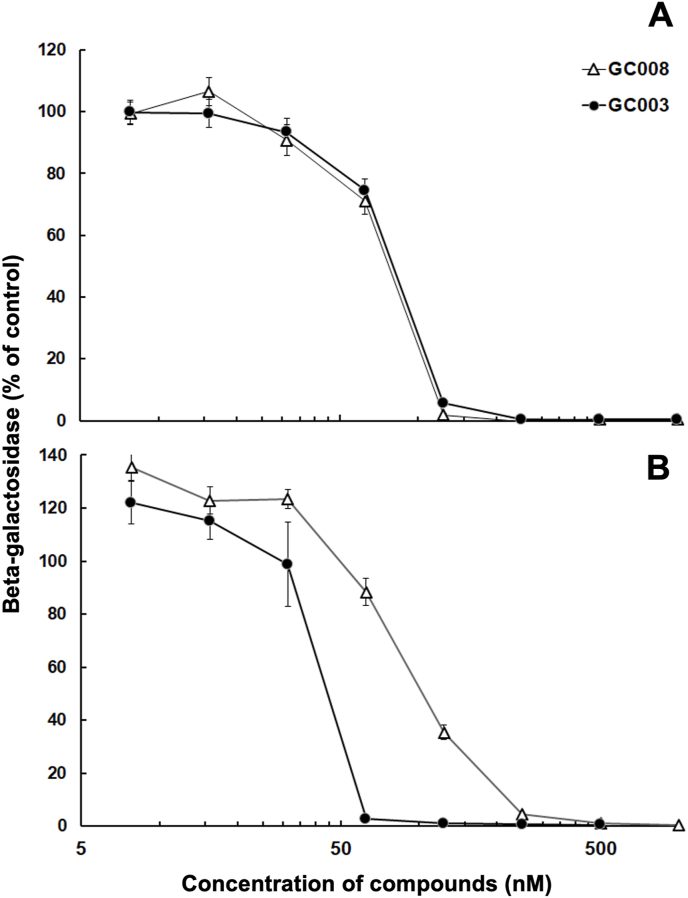
Dose response curves of artemisone (GC003) and artemiside (GC008) added prior to infection (A) or 3 h post infection (B) of confluent human foreskin fibroblasts by tachyzoites of *T. gondii* RH β-galactosidase reporter strain. The mean values ± SE are indicated for quadruplicates.

Drug effects against *T. gondii* tachyzoites were assessed in *vitro* using a transgenic *T. gondii* RH strain constitutively expressing *E. coli* beta-galactosidase. Drugs were added to confluent HFF monolayers either 5 min prior to, or 3 h after infection, with tachyzoites (Fig. 2 A and B, respectively). In both assays, artemisone (GC003) and artemiside (GC008) inhibited the proliferation of *T. gondii* tachyzoites with IC_50_s of approximatively 50–70 nM (Table 1). The host cells were not affected by the compounds in the concentration range tested. Thus, both compounds could be considered as potent inhibitors against *T. gondii* proliferation *in vitro* with IC_50_ values in the sub 10^−7^ M range.

**Table 1:** Inhibition constants (IC_50_ = drug concentration inhibiting proliferation by 50%) of artemisone (GC003) and artemiside (GC008) against *Toxoplasma gondii* RH beta-galactosidase-expressing tachyzoites grown in HFF monolayers as calculated from the data depicted in Fig. 1, and viability impairment of uninfected human foreskin fibroblast host cells. IC_50_ values were calculated using the logit-log algorithm and are indicated with 95% confidence intervals.

	GC003	GC008
*T. gondii* beta-gal at infection	69 [60–80] nM	57 [50–65] nM
*T. gondii* beta-gal 3 h post infection	48 [39–59] nM	76 [67–86] nM
HFF host cells	>2 μM	>2 μM

### 3.2 **Reactive** oxygen **species (ROS) in***T. gondii***tachyzoites and tachyzoite-infected HFF**

**Fig. 3:**
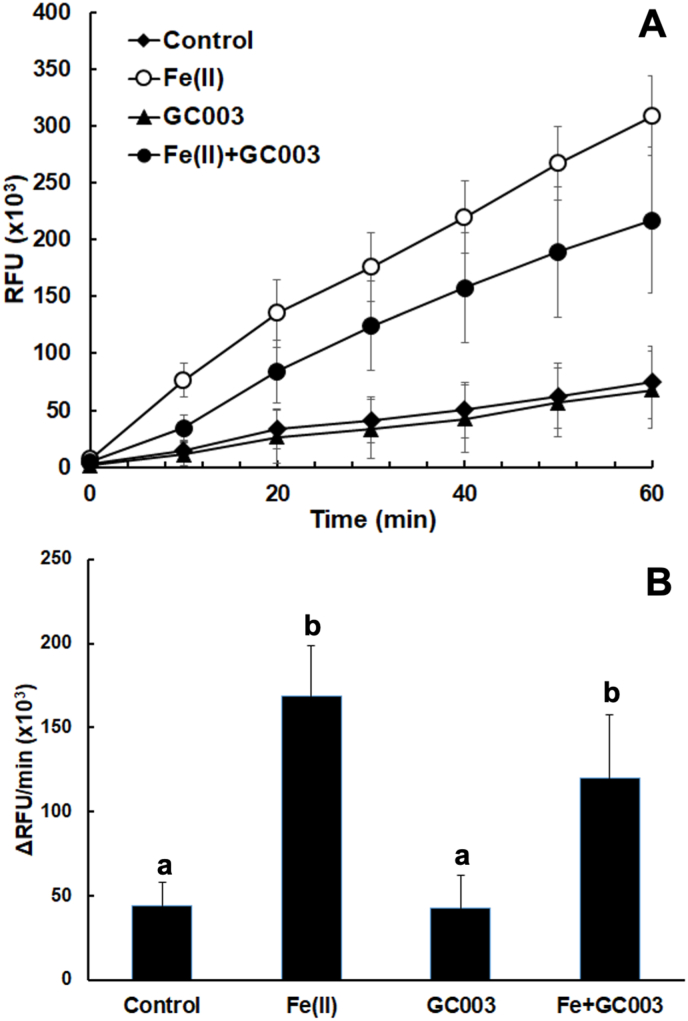
Oxidation of dihydro-dichlorofluorescein to dichlorofluorescein (DCF) by reactive oxygen species of isolated *T. gondii* RH beta-galactosidase tachyzoites. The assay was performed in 96-well-plates containing 10^5^ tachyzoites per well as described in materials and methods in the presence of ferrous ions, artemisone (GC003; 5 μM), both ferrous ions and artemisone, or DMSO as a solvent control. Substrate blanks were subtracted. The mean values ± SE are indicated for eight independent replicates. (A), Formation of DCF during 1 h; (B), initial velocity of DCF formation. Values superscribed by the same letters are not significantly different (p < 0.01; multiple t-tests, corrected for multiplicity of experiments).

In view of the current hypotheses concerning the mode of action of artemisine derivatives, it was tempting to investigate whether the anti-proliferative effects of artemisone, which is formed upon metabolic conversion of artemiside, were correlated to an enhanced ROS formation. Isolated tachyzoites from the same strain as used for the efficacy tests had a basal ROS formation of around 50 relative fluorescent units per min. Addition of 5 μM Fe(II) ions enhanced this rate by a factor of more than three. Conversely, addition of artemisone (GC003) did not enhance ROS formation. When added together with Fe(II), the ROS formation rate was even slightly (but not significantly at p < 0.01) decreased (Fig. 3). In another experiment, ROS formation was assessed in HFF monolayers infected with *T. gondii* tachyzoites and treated during 24 h with artemisone or artemiside. None of these compounds enhanced ROS formation. Artemisone even significantly reduced ROS formation in the presence of Fe(II) (p < 0.01; Table 2).

**Table 2:** Oxidation of dihydro-dichlorofluorescein to dichlorofluorescein (DCF) by reactive oxygen species in confluent HFF monolayers in 96-well-plates infected with 10^3^*T. gondii* RH beta-galactosidase tachyzoites. After 48 h, the cells were treated with artemisone (GC003), artemiside (GC008; 5 μM) or DMSO as a solvent control. After 24 h, the assay was performed as described in materials and methods with or withour ferrous ions. The mean values ± SE, expressed as percentage of the solvent control without ferrous ions, are indicated for eight independent replicates Values superscribed by the same letters are not significantly different (p < 0.01; multiple t-tests, corrected for multiplicity of experiments).

	Without Fe (II)	With Fe(II)
DMSO	100 ± 16.8^a^	194 ± 18.3^b^
Artemisone (GC003)	79.1 ± 8.1^a^	119.9 ± 23.5^a^
Artemiside (GC008)	66.5 ± 14.4^a^	137.1 ± 20.8^b^

### 3.3 **Effects on***T. gondii***ultrastructure**

**Fig. 4:**
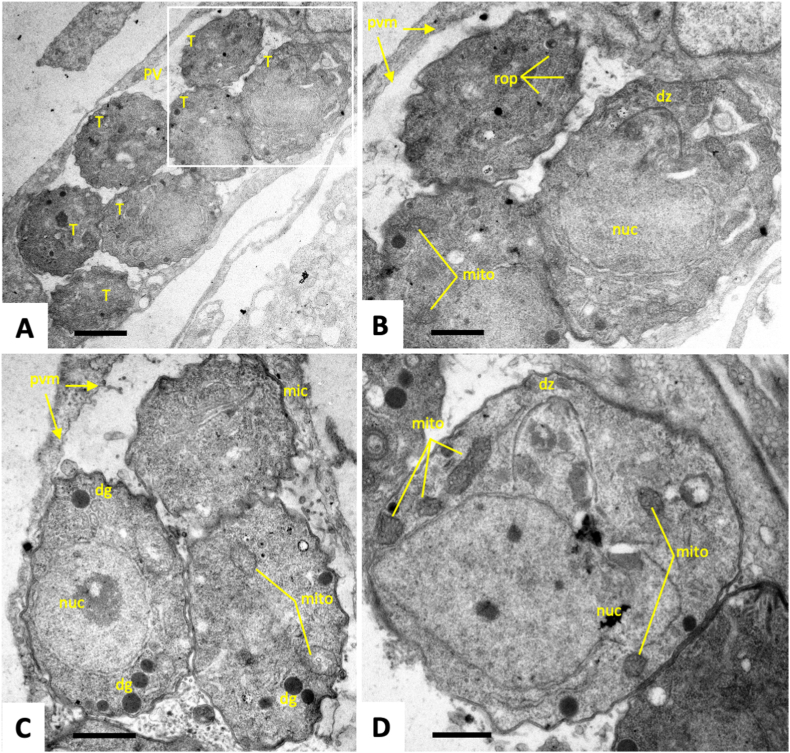
TEM of non-treated *T. gondii* tachyzoites. (A) shows an overview of a parasitophorous vacuole (PV) containing numerous tachyzoites (T). The boxed area in (A) is shown in (B). The parasitophorous vacuole is surrounded by the parasitophorous vacuole membrane (pvm). Parasites undergo cell division by endodyogeny, resulting in the formation of daughter zoites (dz), also shown in (D). Parts of the mitochondrion (mito) are visible as electron dense entities as indicated in (B–D); mic = micronemes, rop = rhoptries, dg = dense granules, nuc = nucleus, pvm = parasitophorous vacuole membrane. Bars in (A) = 2 μm; in (B, C) = 1 μm; in (D) = 0.5 μm).

**Fig. 5:**
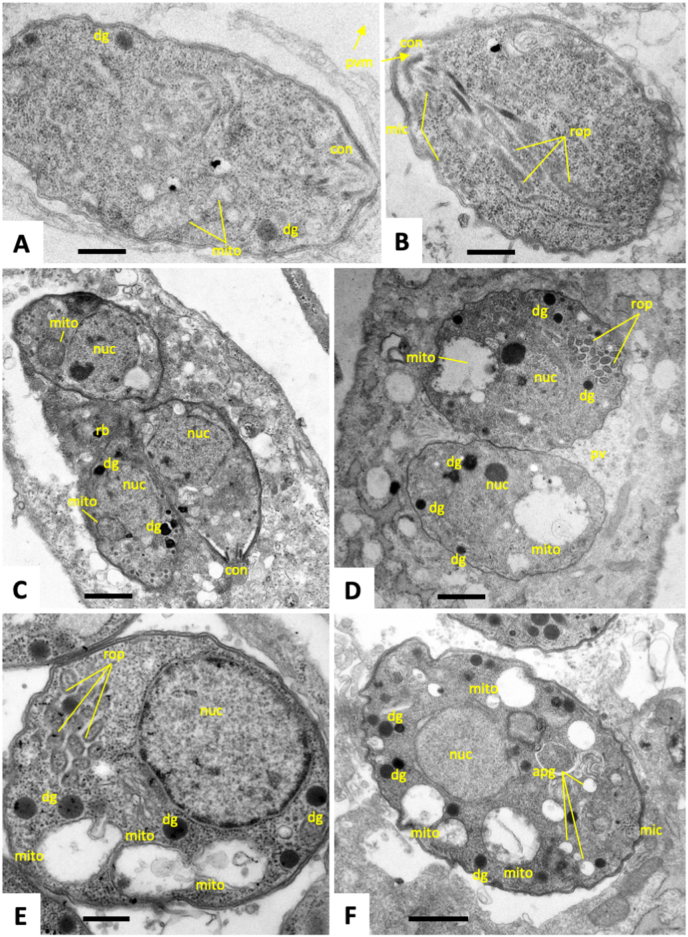
TEM of *T. gondii* tachyzoites cultured in human foreskin fibroblasts and treated with artemisone (GC003) during 6 h (A, B), 24 h (C, D) and 48 h (E, F). After 6 h no ultrastructural changes are seen, except that the mitochondrial matrix (mito) appears to show first signs of desintegration (A, B). (C) Parasitophorous vacuole containing tachyzoites still attached to a residual body (rb) undergoing endodyogeny. (D) Vacuole containing tachyzoites exhibiting large vacuoles, residues of the mitochondrion lacking an intact matrix (mito). (E) Tachyzoite exhibiting similarly altered mitochondrion (mito). (F) Parasite with altered mitochondria as well as inclusions that resemble amylopectin granules (apg); con = conoid, mic = micronemes, rop = rhoptries, dg = dense granules, nuc = nucleus. Bar in (A, B) = 0.5 μm; (C) = 1.5 μm; (D) = 1 μm; (E) = 0.5 μm; (F) = 1 μm.

**Fig. 6:**
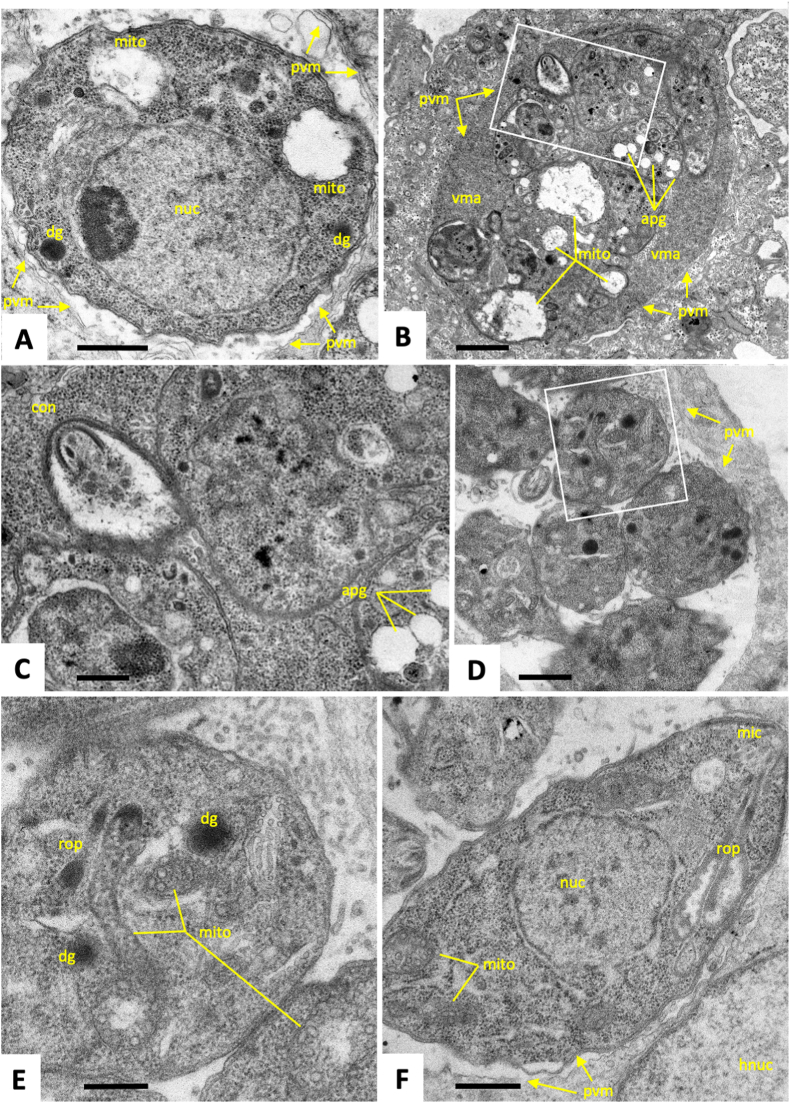
TEM of *T. gondii* tachyzoites cultured in human foreskin fibroblasts and treated with artemiside (GC008) during 24 h (A–C) and 48 h (D–F). Boxed areas in (B) and (D) are enlarged in (C) and (E), respectively. Note the occurrence of mitochondrial damage induced at 24 h (A, B), but not evident anymore at 48 h (D–F); con = conoid; pvm = parasitophorous vacuole membrane; mito = mitochondrion, vma = vacuolar matrix; apg = amylpectin granules; nuc = nucleus; hnuc = host cell nucleus. Bar in (A) = 0.5 μm; (B) = 2 μm; (C) = 0.5 μm; (D) = 2 μm; (E) = 0.5 μm; (F) = 0.7 μm.

TEM was used to visualize potential effects of treatment of with artemisone (GC003) and artemiside (GC008), both applied at 1 μM for a period of 48 h. In non-treated cultures (Fig. 4), tachyzoites proliferated intracellularly within parasitophorous vacuoles surrounded by a parasitophorous vacuole membrane (Fig. 4 A, B). Tachyzoites contain a single mitochondrion, composed of branched tubules (Fig. 4 C). However, only parts of the mitochondrion were visible in any given section plane, displaying a matrix with high electron density (best seen in Fig. 4 D). Tachyzoites divide by endodyogeny, which results in the formation of daughter zoites emerging from larger parent zoites (Fig. 4 B, D). Treatment with artemisone (Fig. 5) for 6 h did not result in any dramatic structural alterations (Fig. 5 A, B). However, in cultures treated for 24 h, a mixed population of tachyzoites was discernible (Fig. 5 C, D). On one hand, structurally largely unaltered parasites were visible. On the other hand, many parasites exhibited “empty” mitochondrial residues, now visible as vesicles that had lost the characteristic electron dense matrix. At 48 h of artemisone treatment (Fig. 5E and F), all parasites had formed larger cytoplasmic vesicles that sometimes contained membranous material, indicating that some of these large vesicles could be mitochondrial residues. In addition, an increased number of amylopectin granules were visible (Fig. 5 F). Overall, other organelles did not seem to be structurally impacted by drug treatments. Treatment with artemiside during 6 h did not lead to obvious structural changes (data not shown), but treatments for 24 h had an impact in that, similar to artemisone, in many parasites the mitochondrion had lost its matrix and the cytoplasm appeared highly vesiculated (Fig. 6 A). A number of parasitophorous vacuoles were detected that contained a highly electron dense vacuolar matrix in which tachyzoites were embedded. These intravacuolar tachyzoites also exhibited aberrant mitochondria, but in addition also many amylopectin granules were seen to be present within the cytoplasm (Fig. 6 B, C). At 48 h, however, larger parasitophorous vacuoles containing numerous tachyzoites were found (Fig. 6D), but these tachyzoites appeared structural much more intact (Fig. 6 E, F), with intact mitochondrial matrix, and secretory organelles. Overall, artemisone and artemiside induce structural alterations, especially in the mitochondrion, with artemisone (GC003) causing more dramatic effects.

### 3.4 Long-term treatments and resistance formation

**Fig. S1:**
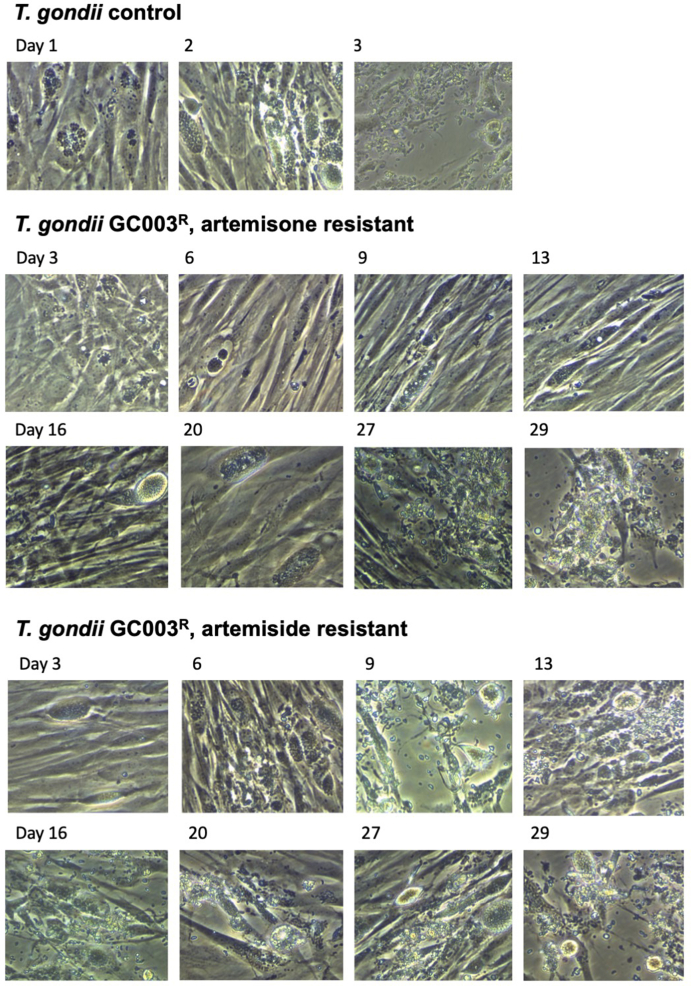
Light microscopy of HFF cultures infected with *T. gondii* ME49 tachyzoites during resistance formation against artemisone (GC003) or artemiside (GC008). Starting concentration for both compounds was 1 μM. Due to a lower growth rate, GC003 concentration was increased to 2.5 μM on day 23 and 5 μM on day 27. GC008 concentration was increased to 2.5 μM on day 13, and further increased to 5 μM on day 23. Resistant parasites undergoing rapid proliferation were collected on day 29. In the absence of drugs, the resistant strain GC003^R^ needed ca. two weeks to completely infect HFF cultures, wheres GC008^R^ and the corresponding wildtype needed only one week.

In order to generate artemisone and artemiside resistant strains, *T. gondii* ME49 infected HFF were exposed to high concentrations of GC003 and GC008, respectively, for a period of 29 days. As a solvent control, one flask was treated with DMSO (0.25%). During the whole treatment period, cultures were monitored light microscopically, as seen in suppl. Figure 1. During the first 20 days of treatment with 1 μM artemisone (GC003), *T. gondii* remained largely intracellular and formed parasitophorous vacuoles. On day 23, lysis of host cells started for the first time. The drug concentration was increased to 2.5 μM, and on day 27 to 5 μM. However, tachyzoite proliferation continued. The same procedure was followed with cultures treated with artemiside (GC008). However, the artemiside concentration had to be increased to 2.5 μM already on day 13. A further increase to 5 μM added on day 23 did not halt parasite proliferation. As a result, the artemisone resistant strain GC003^R^ and the artemiside resistant strain GC008^R^ were generated within 29 days, as depicted in the supplemental Figure S1. The resulting resistant strains keep their resistance over multiple subcultures in the absence of drugs.

In a next step, the drug susceptibilities of these strains were compared to their respective wildtype. Minimal inhibitory concentrations (MIC) of both strains were higher than those of the wildtype strain. In the case of strain GC003^R^, the exact MIC could not be determined since at the highest concentrations employed in this assay (12.5 and 25 μM), the host cells started to lyse. Moreover, both strains had higher IC_50_s than the corresponding wildtype on both drugs. Whereas, the IC_50_s where only slightly higher for strain GC008^R^ than for the wiltype, they were more than one magnitude higher for strain GC003^R^ (Table 3).

**Table 3:** Inhibition constants (IC_50_ = drug concentration inhibiting proliferation by 50%; MIC, minimal inhibitory concentration) of artemisone (GC003) and artemiside (GC008) against *Toxoplasma gondii* ME49 grown in HFF monolayers as determined by quantitative PCR. IC_50_ values were calculated using the logit-log algorithm and are given with 95% confidence intervals, MICs are presented in brackets.

Strain	Artemisone	Artemiside
ME49 wildtype	0.12 (0.06–0.24) μM [0.78 μM]	0.18 (0.15–0.22) μM [1.56 μM]
GC003^R^	1.84 (1.38–2.44) μM [>12.5 μM]	>5 μM [>12.5 μM]
GC008^R^	0.3 (0.20–0.46) μM [6.25 μM]	0.3 (0.2–0.6) μM [6.25 μM]

### 3.5 Proteomic analysis of resistant strains

**Fig. 7:**
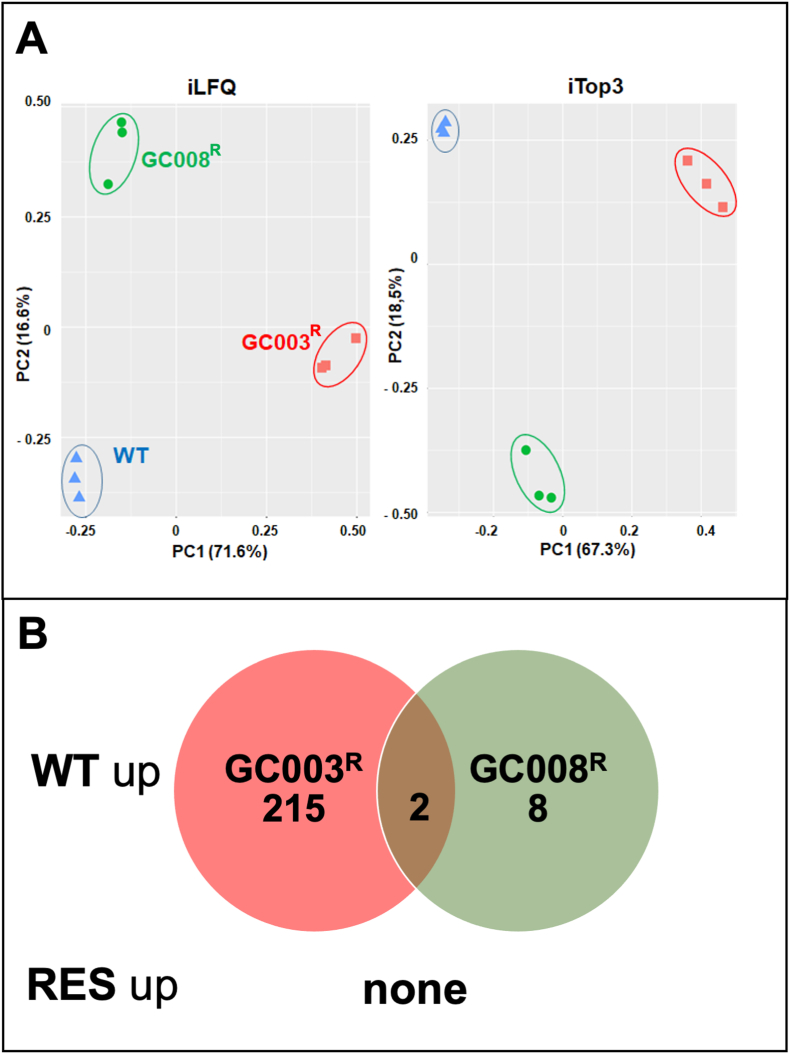
Schematic representation of differential proteome data sets from artemisone (GC003^R^) and artemiside (GC008^R^) resistant and *T. gondii* ME49 wildtype (WT) tachyzoites. (A) Principal component analysis of differential proteomes analyzed via iLFQ and iTOP3 algorithms; (B) Venn diagram detailing the number of differentially expressed proteins (by both algorithms). Tachyzoites of resistant strains (GC003^R^, red symbols; GC008^R^, green symbols) were compared to their wildtype (blue symbols) by MS shotgun analysis as described in Materials and Methods.

**Fig. 8:**
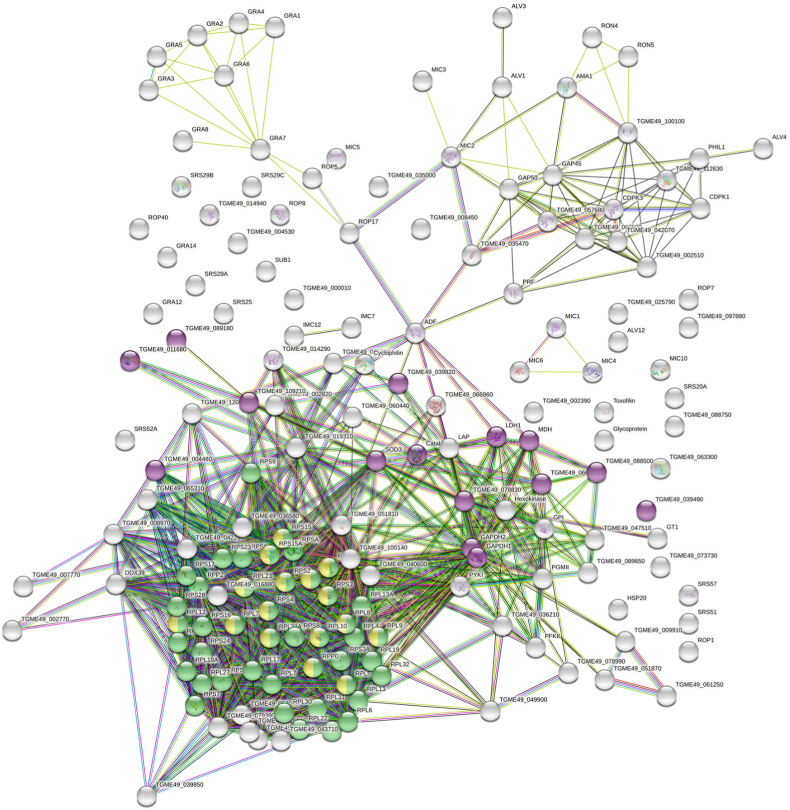
Protein-protein interaction network of proteins differentially downregulated in GC003^R^ tachyzoites compared to *T. gondii* wildtype tachyzoites. Oxidoreductases (pink) and structural components of ribosomes (green) including rRNA binding proteins (yellow) are highlighted. The interaction network was created by the STRING knowledgebase and software tool from the Swiss Institute of Bioinformatics (www.expasy.org).

Differential proteomic analysis of the artemisone (GC003^R^) and artemiside (GC008^R^) resistant strains versus their corresponding *T. gondii* ME49 wildtype yielded 3977 unique peptides matching to 733 *T. gondii* proteins. The complete dataset is available as supplemental Table S1 under https://doi.org/10.5281/zenodo.7101485. Overall analysis of the data by principal component analysis revealed three non-overlapping clusters of wildtype, GC003 and GC008 resistant strains by both iLFQ and iTop3 algorithms (Fig. 7 A). A more detailed analysis revealed that 215 proteins were significantly downregulated in GC003^R^ and 8 proteins in GC008^R^ as compared to their wildtype ME49. Two proteins were downregulated in both strains. No proteins were upregulated in the resistant strains as compared to their corresponding wildtype (Fig. 7 B). The complete list of the differentials is given as supplemental Table S2 (https://doi.org/10.5281/zenodo.7101485). The high number of differentials between wildtype and GC003^R^ prompted us to analyze the protein–protein interaction network generated by the STRING knowledge base and software tool (Swiss Institute of Bioinformatics). The knowledge base could map 194 of the 217 differentials yielding 168 nodes with 1861 edges. This number is significantly higher (p < 10^−16^) than the expected 838 edges. Thus, the differentially downregulated proteome clearly comprises clusters. The two most prominent clusters, namely ribosomal components comprising 39 proteins and 15 oxidoreductases, are highlighted in Fig. 8.

Moreover, key enzymes of glycolysis such as phosphofructokinase, fructose-1,6-bis-phosphate aldolase, glyceraldehyde-3-phosphate dehydrogenase and others were amongst the downregulated proteins in strain GC003^R^ (Table S2).

The ten significantly downregulated proteins in strain GC008^R^ comprised a ribosomal protein, three hypothetical proteins, two rhoptry neck proteins, one rhoptry protein, the inner membrane complex protein IMC2A, and a redoxin-domain containing protein (Table 4).

**Table 4:** List of the differentially downregulated proteins in *Toxoplasma gondii* tachyzoites from the artemiside resistant strain GC008^R^ as compared to its wildtype ME49. The proteins marked by an asterisk are downregulated in strain GC003^R^, as well.

ORF in ToxoDB	Annotation
TGME49_207170	Hypothetical protein
TGME49_223050	Ribosomal protein RPS20
TGME49_228170	Inner membrane complex protein IMC2A
TGME49_236950	Hypothetical protein*
TGME49_286630	Redoxin domain-containing protein
TGME49_290700	Hypothetical protein
TGME49_300100	Rhoptry neck protein RON2*
TGME49_306060	Rhoptry neck protein RON8
TGME49_312600	Heat shock protein HSP21
TGME49_315490	Rhoptry protein ROP10

**Fig. 9:**
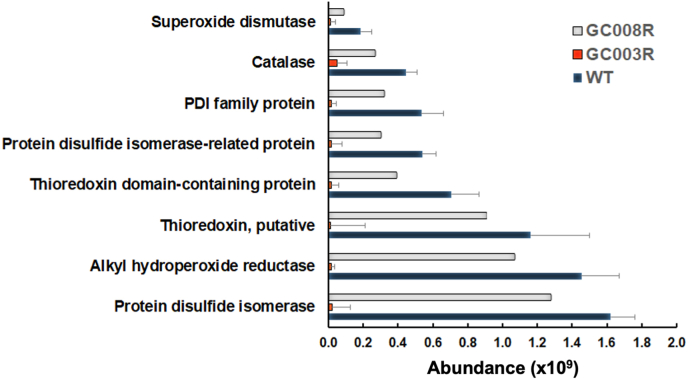
Quantitative assessments of proteins involved in oxidative stress response. Tachyzoites of artemisone (GC003^R^) and artemiside (GC008^R^) resistant strains were subjected to MS shotgun analysis as described in Materials and Methods. For all proteins, mean values ± standard errors for iTop3 intensities in three biological replicates are shown. The differences were statistically significant in strain GC003^R^, but not in strain GC008^R^. See Table S1 for complete list and statistics.

In relation to proteins involved in ROS scavenging, eight proteins including protein disulfide isomerases, superoxide dismutase and catalase were significantly downregulated in GC003^R^ vs wildtype. In GC008^R^ tachyzoites, their intensities were also lower, but the differences to the wildtype were not significant (Fig. 9).

**Fig. 10:**
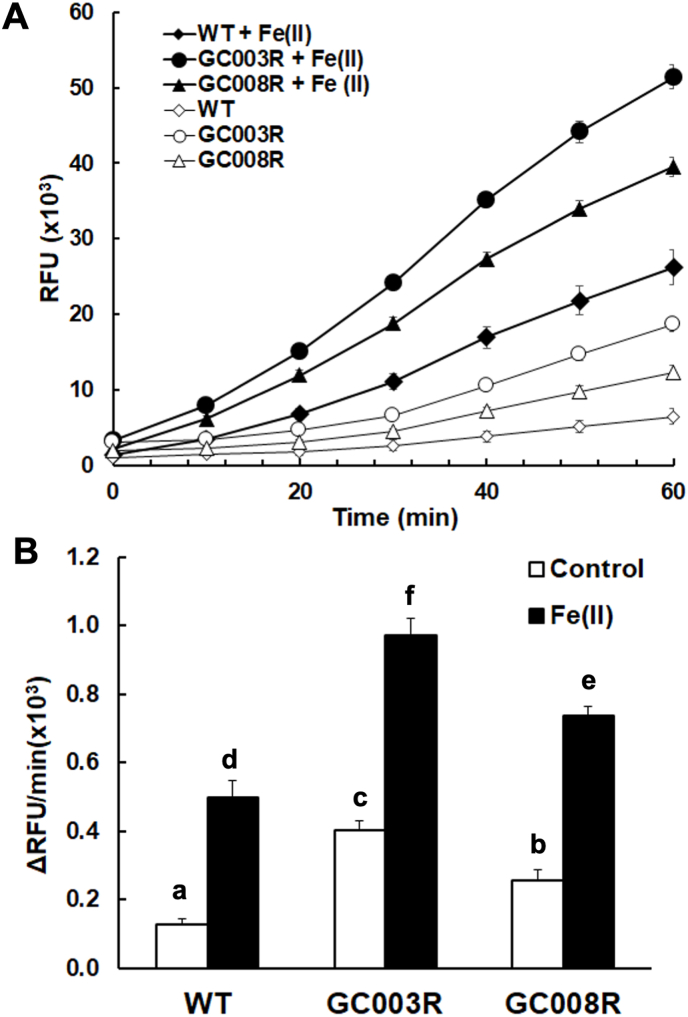
Oxidation of dihydro-dichlorofluorescein to dichlorofluorescein (DCF) by reactive oxygen species of isolated *T. gondii* tachyzoites from artemisone (GC003) and artemiside (GC008^R^) resistant strains and from their corresponding wildytype (WT). The assay was performed in 96-well-plates containing 10^4^ tachyzoites per well as described in materials and methods with or without ferrous ions (5 μM) in the buffer. Substrate blanks were subtracted. The mean values ± SE are indicated for eight replicates. (A), Formation of DCF during 1 h; (B), initial velocity of DCF formation. Values superscribed by the same letters are not significantly different (*p* < 0.002; multiple t-tests, corrected for multiplicity of experiments).

These findings prompted us to compare the ROS formation in isolated tachyzoites from both resistant strains to tachyzoites of the corresponding wildtype ME49. In tachyzoites from all strains, after a lag phase of ca. 20 min, ROS formation steadily increased over more than 1 h and was higher in tachyzoites from both resistant strains than in wildtype tachyzoites with or without Fe(II) ions (Fig. 10 A). This observation is fostered by comparing the initial velocities of ROS formation after the lag phase. In isolated tachyzoites from GC003^R^, ROS formation was significantly faster than in GC008^R^ tachyzoites, which in turn had an enhanced ROS formation as compared to wildtype tachzoites. The presence of Fe(II) ions significantly enhanced ROS formation in tachyzoites from all strains without altering with their ranking (Fig. 10 B).

One of the two proteins downregulated in both strains was the Rhoptry neck protein RON2, a 1479 amino acid protein encoded by ORF TGME49_300100. The other downregulated protein was a hypothetical protein encoded by ORF TGME49_236950. This 121 amino-acid protein – highly conserved amongst other apicomplexans – has no homologies to other known proteins. To identify a possible function of this protein, a modeling was performed by comparing a potential structure to existing structures sharing similarities. In the Swiss Model repository, the template with the highest identity (QMEANDisCo Global value 0.35 ± 0.12) was 3zyy.1, the reductive activator of corrinoid iron-sulfur proteins from *Carboxydothermus hydrogenoformans* (Hennig et al., 2012).

## 4 Discussion

In our study, artemiside (GC008) and artemisone (GC003) exhibit promising *in vitro* efficacies against *T. gondii* with a broad therapeutic index thereby confirming previously published results (Dunay et al., 2009). In terms of drug-induced ultrastructural changes, the treatments of infected cells with both compounds at 1 μM resulted in alterations within the cytoplasm of tachyzoites. The compounds appeared to mainly impact on the mitochondria, the matrix of which is clearly discernible in control cultures by the characteristic electron dense pattern formed by tightly packed cristae, but not in drug-treated cultures. Moreover, the lumen of the mitochondria has increased in size upon drug treatments. Alterations were not seen in parasites after 6 h of drug treatments, but were most evident at 24 h. The loss of mitochondria was associated in parallel with the occurrence of cytoplasmic vesiculation, and in some instances, cytoplasmic vesicular inclusions were seen to contain membranous residues that resembled cristae. This indicated that many of these inclusions were in fact mitochondria largely lacking their content. The mitochondrial membranes, however, appeared to remain structurally undisturbed. Similar changes in mitochondrial structures were seen earlier in *T. gondii* tachyzoites treated with drugs that also target the mitochondrion, such as decoquinate (Ramseier et al., 2021), or organometallic ruthenium complexes (Anghel et al., 2021b), and in other related apicomplexans such as *Neospora caninum* and *Besnoitia besnoiti* treated with endochin-like quinolones and buparvaquone, respectively (Müller et al., 2019; Eberhard et al., 2020; Anghel et al., 2021a). Overall, drug-induced alterations were more severe for artemisone than for artemiside, and in several instances parasites in artemiside (GC008) treated cultures appeared to recover, and exhibited a structurally intact mitochondrion at 48 h, while for artemisone (GC003) these changes persisted.

These effects seen in these parasites, however, are not linked to a differential formation of reactive oxygen species (ROS). In the presence of artemisone, ROS formation not only not increased, but even slightly decreased, in isolated tachyzoites, and the same was observed in infected host cells, as well as in uninfected cells (data not shown). This may be due to a complexation of ferric ions, as previously described (Haynes et al., 2013).

By long term treatments of *T. gondii* infected HFF in the presence of increased concentrations, two cell lines (named GC003^R^ and GC008^R^) were generated. Since in the presence of both artemisone and artemiside, both cell lines had elevated MICs and not only elevated IC_50_s as compared to the wildtype, they were regarded as “resistant” to both compounds, in analogy to what is defined with respect to antibiotic resistance in bacteria (Brauner et al., 2016). The resulting resistant strains keep their resistance over multiple subcultures in the absence of drugs. Analysis of their proteomes reveals pronounced differences between artemiside and artemisone resistant lines. Whereas in artemisone resistant GC003^R^ tachyzoites 217 proteins have significantly lower levels than in wildtype tachyzoites, the corresponding number in artemiside resistant GC008^R^ tachyzoites is only 10. Only two proteins are commonly downregulated in both datasets. In a previous study on artemisinin resistant strains of *T. gondii*, point mutations in a protease and a protein kinase – both with unknown functions –, as well as an amplification of the mitochondrial genome are correlated with resistance or susceptibility (Rosenberg et al., 2019). None of the proteins encoded by the mutated genes in this study are amongst the differentials in our dataset, nor do we have evidence that enzymes involved in heme metabolism as identified in DHA resistant strains (Harding et al., 2020) are involved in artemisone or artemiside resistance. Both compounds are amino-artemisinins and have a higher effectiveness against *T. gondii* than artemisinin. It is therefore questionable whether a direct comparison of resistance mechanisms can be made.

Concerning the artemisone resistant strain GC003^R^, many of the downregulated proteins are involved in protein biosynthesis and intermediary metabolism, suggesting a transition from active growth to a dormant state. This is further corroborated by the appearance of the amylopectin granules, and by a slower growth rate as compared to the wildtype. This transition may be triggered by an altered calcium homeostasis, as observed in artemisinin resistant *T. gondii* mutants (Nagamune et al., 2007a, 2007b). Similarly, in *Plasmodium* sp. resistant to artemisinin, a phenomenon called “quiescence” correlated to lower protein biosynthesis has been observed (Witkowski et al., 2010). Whole-genome sequencing of artemisinin resistant strains correlates dominant mutations in the gene encoding for the Kelch-13-propeller domain protein to artemisinin resistance (Ariey et al., 2014). Based on analogy to the human Keap1 complex involved in oxidative stress response, a current model suggests that in the mutant strains, the unfolded protein response pathway (Hetz, 2012) is upregulated resulting in cell cycle arrest (Paloque et al., 2016). The observed downregulation of proteins involved in scavenging of reactive oxygen species (ROS) may thus be a consequence of a general metabolic shutdown by the artemisone resistant strain, in particular. This explains why this strain produced more ROS then the wildytype strain and the artemiside resistant strain, where only a minor downregulation of the respective proteins was observed.

The two proteins downregulated in both artemisone and artemiside resistant strains, the rhoptry neck protein RON2, and the hypothetical protein TgME49_236950 thus far have not been investigated with respect to drug modes of action. RON2 is known to interact with the apical membrane protein AMA1 forming a complex playing a crucial role in host cell invasion (Lamarque et al., 2014). It is unclear whether this protein has other functions. The hypothetical protein TgME49_236950 shares some similarities with redox activators of prokaryotic corrinoid proteins. Corrinoids are cobalt-containing cofactors involved in methyl-transfer processes such as methanogenesis (Matthews, 2009). An example for corrinoids in eukaryotes is cobalamin (vitamin B12). This hypothetical protein is highly conserved in apicomplexans. Its function, however, is unknown. Its loss in resistant strains suggests a gain of function with respect to drug interaction, such as drug activation, for instance. Another possibility could be inhibition of metabolic repression. The investigation of this function and search for interacting proteins in *Toxoplasma gondii* and related apicomplexans necessitates further investigation.

## Author contributions

J.M., C.S., conception of the study, functional assays, writing; C.S., generation of resistant lines; M. H., S.B.-L., A.-C. U, proteomics; R. K. H., A. H. electron microscopy, writing, conception, supervision, funding.

## Funding

This work was funded by the 10.13039/100000001Swiss National Science Foundation project 310030_184662, and 10.13039/501100001322the South African Medical Research Council (MRC) Flagship Project MALTB-Redox with funds from 10.13039/501100016979the National Treasury under its Economic Competitiveness and Support Package (UID MRC-RFA-UFSP-01–2013) (RKH), and by 10.13039/100012106the South African National Research Foundation (SASA NRF) grant (UID 129135) (RKH).

## Declaration of competing interest

The authors declare that the research was conducted in the absence of any commercial or financial relationships that could be construed as a potential conflict of interest.

## Appendix A Supplementary data

Table S1 List of proteins identified in *Toxoplasma gondii* ME49 wildtype, artemisone (GC003^R^), and artemiside (GC008^R^) resistant tachyzoites. Table S1

Table S2 List of proteins downregulated in strain GC003^R^; Table S2
